# Simvastatin reduces circulating oxysterol levels in men with hypercholesterolaemia

**DOI:** 10.1016/j.redox.2018.02.014

**Published:** 2018-02-17

**Authors:** Irundika H.K. Dias, Ivana Milic, Gregory Y.H. Lip, Andrew Devitt, M. Cristina Polidori, Helen R. Griffiths

**Affiliations:** a Aston Research Centre for Healthy Ageing, School of Life and Health Sciences, Aston University, Birmingham B4 7ET, UK; b Institute of Cardiovascular Sciences, University of Birmingham, Birmingham, UK; c Institute of Biochemistry and Molecular Biology I, Heinrich-Heine-University, Duesseldorf, Germany; d Ageing Clinical Research, Department Medicine II, University Hospital of Cologne, Cologne, Germany; e Faculty of Health and Medical Sciences, University of Surrey, Stag Hill, Guildford GU2 7XH, UK

**Keywords:** 24S-OHC, 24S-hydroxycholesterol, 25-OHC, 25-hydroxycholesterol, 27-OHC, 27-hydroxycholesterol, 7β-OHC, 7β-hydroxycholesterol, 7-KC, 7-ketocholesterol, AD, Alzheimer's disease, ApoE, Apolipoprotein E, BHT, Butylated hydroxytoluene, oxLDL, oxidised low density lipoprotein, CE, Collision Energy, CH25H, Cholesterol 25-hydroxylase, CV, Coefficient of variation, CXP, exit quadrapole potential, DP, Declustering potential, ELISA, Enzyme linked Immunosorbent assay, ESI, Electro spray Ionisation, GC, Gas chromatography, LC-MS/MS, Liquid Chromatography Mass spectrometry, LLOQ, lower limits of quantification, MRM, Multiple reaction monitoring method, PR, Process Recovery, QC, Quality control, SD, Standard deviation, SE, Standard error, SPE, Solid phase extraction, Hydroxycholesterol, Mass spectrometry, Statin, Vascular disease, Dementia

## Abstract

Oxysterols (OHC) are biologically active cholesterol metabolites circulating in plasma that may be formed enzymatically (e.g. 24S-OHC, 25-OHC and 27-OHC) or by autoxidative mechanisms (e.g. 7-ketocholesterol, 7β-OHC and 25-OHC). Oxysterols are more soluble than cholesterol and are reported to exert inflammatory, cytoprotective and apoptotic effects according to concentration and species. Esterified oxysterols have been analysed in people with dementia and cardiovascular diseases although there is no consistent relationship between oxysterol esters and disease. However, oxysterol esters are held in lipoprotein core and may not relate to the concentration and activity of plasma free oxysterols. Methodological limitations have challenged the analysis of free oxysterols to date.

We have developed a fast, sensitive and specific quantitative LC-MS/MS, multiple reaction monitoring (MRM) method to target five oxysterols in human plasma with analyte recoveries between 72% and 82% and sensitivities between 5 and 135 pg/ml. A novel method was used to investigate the hypothesis that simvastatin may reduce the concentrations of specific plasma free oxysterols in hypercholesterolaemia.

Twenty healthy male volunteers were recruited (aged 41–63 years); ten were asymptomatic with high plasma cholesterol > 6.5 mM and ten were healthy with normal plasma cholesterol (< 6.5 mM). Simvastatin (40 mg/day) was prescribed to those with hypercholesterolaemia. Plasma samples were taken from both groups at baseline and after three months. Simvastatin reduced plasma cholesterol by ~35% (p < 0.05) at the end of three months.

Oxysterols generated by autoxidation (but not enzymatically) were elevated up to 45 fold in hypercholesterolaemic midlife men. Plasma oxysterols were restored to those of healthy controls after simvastatin intervention suggesting that autoxidation is either prevented by simvastatin directly or that autoxidation is less prevalent when plasma cholesterol concentrations are within the normal range.

## 1 Introduction

The epsilon 4 allele of apolipoprotein E (ApoE) remains the strongest genetic risk factor for dementia [1], [2]; it is the lowest affinity ApoE isoform for cholesterol uptake by the lipoprotein receptor. Several modifiable vascular risk factors in midlife are also associated with the development of dementia decades later, including smoking and hypercholesterolaemia [3], [4], [5]. In addition, independent studies have confirmed that statins are effective at reducing the risk for dementia in later life by 25–50% [6], [7]. These observations have led to the suggestion that modification of cholesterol metabolism in midlife may reduce later risk for dementia. However, plasma cholesterol is frequently not elevated in dementia. Instead oxidised lipids have been proposed to be more pathogenic molecules. In support of this, we have previously shown that oxidised low density lipoprotein (oxLDL) is associated with impaired cognition in Alzheimer's disease (AD), the most common form of dementia [8], [9]. Furthermore, lipids extracted from oxLDL are pro-oxidant, neurotoxic and pro-inflammatory in a blood-brain barrier model [10], [11].

Oxysterols are oxygenated derivatives of cholesterol formed by endogenous enzymatic reaction or non-enzymatic auto-oxidation caused by free radicals [12]. These 27-carbon oxidised derivatives are present in very low concentrations in plasma, tissues and cells compared to cholesterol. All oxysterols have a similar chemical structure; a tetracyclic cyclopentaphenanthrene with an isooctyl side-chain at C17 and a hydroxyl group at C3 - the difference between distinct oxysterols lies in the addition of an extra hydroxyl, oxo, keto or epoxy group into the ring structure or to the side chain [12]. These characteristics make it challenging to analyse low concentrations of oxysterols. However, advances in mass spectrometry (MS)-based approaches have enabled the identification and quantification of oxysterols in biological samples.

24S-hydroxycholesterol (24S-OHC) is formed enzymatically in a subset of neurons in the brain by cholesterol 24-hydroxylase, a cytochrome P450 (CYP46A1) enzyme that convert cholesterol to 24S-OHC [13]. 24S-OHC regulates cholesterol homeostasis and supports neuronal function through activation of liver X receptors [14]. The increase in plasma 24S-OHC in plasma during dementia is thought to reflect loss of neurones and increased transport to the periphery. 27-hydroxycholesterol (27-OHC) is formed in the liver by the sterol 27-hydroxylase CYP27A1 and may be exported into the brain. *In vitro* studies suggest that 27-OHC has concentration-dependent neurotoxic and neuroprotective properties [11], [14], [15]. Investigations into variations in plasma 27-OHC concentrations with dementia have proved inconclusive [11]. However, recent studies report an increase in plasma 27-OHC during mild cognitive impairment [16] and peripheral artery disease [17].

The most abundant free radical-dependent autoxidation products in plasma, plaques and tissues are 7-ketocholesterol (7-KC) and 7β-hydroxycholesterol (7β-OHC) [12]. Autoxidised sterols have been shown to modify gene expression in endothelial cells, affect angiogenesis, inflammation and are present in high concentrations in atheromatous plaques [18], [19]. 25-hydroxycholesterol (25-OHC) may be formed by the enzyme cholesterol 25-hydroxylase (CH25H), induced by lipopolysaccharide or type I interferon after bacterial or viral infection and by autoxidation [20]. It is found at elevated concentrations in plaques and in plasma during vascular disease, is pro-inflammatory via activation of Toll-like receptors and modulates sterol metabolism via SREBP2 [17], [21], [22], [23].

Hypercholesterolaemia in midlife is a risk factor for dementia in later life, associates with increased oxysterol concentration, and is ameliorated by statins. Statins have been reported to lower plasma esterfied oxysterol concentration [24], as (non-specifically) analysed using gas chromatography (GC) or by non-specific enzyme linked immunosorbent assay (ELISA) [25]. Therefore, we have developed a more specific and sensitive MS-based method for quantification of five biologically active, non-esterfied oxysterols namely 24S-OHC, 25-OHC, 27-OHC, 7-KC and 7β-OHC in plasma in a single analytical run. Using this advanced method, we have investigated the hypothesis that simvastatin treatment in hypercholesterolaemia may reduce the concentrations of specific plasma oxysterols.

## 2 Materials and methods

### 2.1 Chemicals

Authentic standards (24(S)-hydroxycholesterol, 27-hydroxycholesterol, 25-hydroxycholesterol, 7ß-hydroxycholesterol) and deuterated (24(R/S)-hydroxycholesterol-d7, 25-hydroxycholesterol-d6, 27-hydroxycholesterol-d6, 7ß-hydroxycholesterol-d7, 7-ketocholesterol-d7) were purchased from Avanti polar lipids, Alabama. Authentic standard 7 keto cholesterol was purchased from Cayman chemicals, MI, USA. Butylacetate, hexane, isopropanol, methanol and formic acid (HPLC/MS grade) were purchased from Fisher Scientific, UK. Butylated hydroxytoluene (BHT) and Discovery DSC18 cartridges were from Sigma-Aldrich, UK. Oasis HLB Prime and Oasis HLB cartridges were purchased from Waters.

### 2.2 Plasma sample preparation

Twenty midlife, cardiovascular symptom-free male adults (40–60 years old, mean age 46.9 years) were recruited from general medical practices in the Birmingham area with (total cholesterol > 6.5 mM measured; n = 10) and without (n = 10) hypercholesterolaemia as described in our previous publication [10]. The patient demographics are described in Table 1. All ten statin-naïve, hypercholesterolaemic subjects were prescribed simvastatin intervention (40 mg/day), whereas normolipidaemic subjects maintained habitual diets and lifestyles without intervention. Patients were re-sampled after 3 months. All ten hypercholesterolaemic patients complied with the intervention for the study duration of 3 months. The research was carried out in accordance with the Declaration of Helsinki (2008) of the World Medical Association and ethical approval was obtained from the Birmingham and Black Country Local Research Ethics Committee (REC 09/H1202/87). Participants provided informed written consent.

**Table 1:** Demographics of healthy control and hypercholesterolaemic patients at baseline and 3 month follow up visit. Lipid profiles were determined on the plasma and values are mean ± standard error of mean (SEM); medians and ranges are indicated in parentheses. BMI: body mass index; LDLc: low density lipoprotein cholesterol; HDLc: high density lipoprotein cholesterol. Statistical analysis was performed by two way ANOVA followed by Sidak's comparison: ** and *** indicate statistically significant differences (P < 0.001, P < 0.0001 respectively) between healthy control versus hypercholesterolaemic subjects at baseline.

	Baseline		3 months follow up	
	Control (n = 10)	Hypercholesterolaemic (n = 10)	Control (n = 10)	Hypercholesterolaemic (n = 10)
Weight (Kg)	62 ± 2.47	63.8 ± 2.69	61 ± 2.3	64 ± 2.7
BMI Kg/m^2^	24.88 ± 0.74	26.35 ± 1.1	24.7± 0.68	26.3 ±1.2
Age (years)	46.4 ± 1.7	47.4 ± 1.7	46.4 ± 1.7	47.4 ± 1.7
Cholesterol (mM)	4.08 ± 0.18	6.72 ± 0.78 **	3.8 ± 0.13	4.63±0.31
HDLc (mM)	1.3 ±0.1	1.01 ± 0.07	1.3 ± 0.24	1.28 ± 0.07
LDLc (mM)	1.9 ± 0.17	4.82 ± 0.12 **	1.69 ± 0.41	1.98 ± 0.25
Triglycerides (mM)	1.95 ± 0.3	1.88 ± 0.21	1.67 ± 0.61	1.63 ± 0.18
24S-OHC (ng/ml)	31 ± 4	61 ± 4	39 ± 4	42 ± 3
	(26; 18–62)	(62; 31–86)	(44; 28–47)	(39; 29–60)
25-OHC (ng/ml)	118 ± 32	916 ± 168 **	136 ± 13	120 ± 14
	(154; 76–374)	(171; 46–1845)	(133; 66–185)	(117; 61–207)
27-OHC (ng/ml)	31 ± 2	47 ± 3	39 ± 2	41 ± 2
	(29; 23–43)	(48; 36–60)	(39; 33–51)	(40; 31–49)
7β-OHC (ng/ml)	98 ± 28	4429 ± 762 ***	34 ± 14	23 ± 3
	(60; 14–206)	(4462; 570–7857)	(16; 12–160)	(20; 11–48)
7-KC (ng/ml)	69 ± 18	2302 ± 215 ***	24 ± 9	18 ± 1
	(50; 15–195)	(2343; 826–3176)	(14; 12–104)	(17; 13–27)

Human blood was collected in the EDTA tubes from three healthy individuals and blood plasma was separated by centrifugation for 10 min at 3000 *× g* at 4 °C, collected and pooled to make control for the estimation of matrix effect, recovery and standard curve. Plasmas were aliquoted in 0.5 ml polypropylene tubes and stored at − 80 °C until analysis. Once aliquots were thawed, they were analysed and then discarded.

### 2.3 Extraction of free oxysterols from plasma

We tested enrichment efficiencies of three types of solid phase extraction (SPE) cartridges; Oasis HLB Prime (bed wt. 30 mg, 1 ml volume, Waters), Oasis HLB (bed wt. 30 mg, 1 ml volume, Waters) and Discovery DSC18 (bed wt. 30 mg, 1 ml volume Sigma-Aldrich), for the enrichment of oxysterols from 70 µl of human plasma, spiked with 1 ng of internal standards. Plasmas were mixed with 430 µl methanol, vortexed and incubated on ice for 10 min in the presence of 4 mg/ml BHT before centrifugation at 14,000 × *g* for 10 min.The methanolic supernatant was diluted with acidified water up to 12.5 % of methanol for loading onto an SPE cartridge**.**

SPE cartridges were activated by applying 0.8 ml methanol on the dry bed, followed by the equilibration with 0.8 ml of 1% formic acid in water (v/v, pH~2) using the consistent flow rate of 1 drop/sec. Samples were applied on the wet bed followed by washing with 0.5 ml of 0.1% formic acid in water (v/v, pH~2). The SPE bed was washed with 0.6 ml of hexane to elute hydrophobic lipids. Finally, oxysterols were eluted with 1.8 ml of butyl acetate and collected in 2 ml polypropylene tube. Eluates were dried under vacuum, re-suspended in 20 µl of 50% aqueous methanol containing 0.1% formic acid and analysed immediately.

Oxysterols from healthy and hypercholesterolaemic individuals spiked with deuterated internal standards (1 ng 24-OHCd7, 0.25 ng 25-OHCd6, 4 ng 27-OHCd6, 0.5 ng 7β-OHCd7, 15 ng 7-KCd7) were analysed as previously described. Oxysterols were enriched on Oasis HLB Prime SPE plates (bed wt. 30 mg, 1 ml, 96-well) for a higher throughput.

### 2.4 Estimation of recovery, stability and matrix effects for oxysterol quantification

Pooled plasma from healthy volunteers (male and female) were used for the estimation of oxysterol recovery and stability. Plasma (70 µl) was used with and without spiking with methanolic solution of all authentic and internal standards (0, 0.1, 0.25, 0.5, 0.75, 1, 2.5, 5, 10 ng) in triplicate to calculate the intra- and inter- day recoveries. Stability of the oxysterols during 1–4 freeze-thaw cycles were investigated for one control plasma sample (Table S1). High percentage recovery was observed for all five oxysterols with only one freeze-thaw cycle; 24S-OHC: 75.3%, 25-OHC: 72.6%, 27-OHC: 71.8%, 7β-OHC: 68.2% and 7-KC: 72.7%, compared to 2 or more cycles.

### 2.5 Optimization of detection parameters

Optimization of multiple reactions monitoring (MRM) parameters was performed on a ESI-QqLIT-MS (QTRAP 5500, AB Sciex UK Ltd., Warrington) operated in a positive ion mode with an ionisation voltage of 5.5 kV, entrance potential of 10 V, and ion source temperature of 300 °C. Solutions of authentic and internal standards (100 pg/ml in isopropanol: methanol:water 50:40:10 v/v/v/) with 0.1% formic acid were used for the optimization of collision energy (CE), declustering potential (DP), and exit quadrupole potential (CXP) for each Q1/Q3 (precursor ion/fragment ion) *m*/*z* transition (Table 2). Standard solutions were directly infused into the mass spectrometer for the optimization of Q1/Q3 transition pairs using an integrated syringe pump (Harvard Apparatus) at 10 µl/min flow rate. The final MRM (35 pairs) adopted the three most intense structure specific transitions for each analyte with a dwell time of 55 ms.

**Table 2:** Selected Multiple reaction monitoring (MRM) parameters (Q1/Q3 transition pair; declustering potential (DP); collision energy (CE); exit quadrupole potential, (CXP), retention times) used in the analysis.

	Common name	MRM transitions	DP (V)	CE (V)	CXP (V)	Dwell time (ms)	Retention time (min)
Authentic Standards	24S hydroxycholesterol	385.3/161	166	27	24	55	11.43
	25 hydroxycholesterol	385.3/147	161	33	20	55	11.93
	27 hydroxycholesterol	385.4/161	181	33	14	55	12.88
	7β-hydroxycholesterol	385.4/81	216	53	8	55	13.80
	7-keto-cholesterol	401.4/95	196	41	16	55	14.79
Deuterated Standards	24(R/S)-hydroxycholesterol-d7	392.4/135	196	35	6	55	11.38
	25-hydroxycholesterol-d6	391.6/161	121	33	18	55	11.98
	27-hydroxycholesterol-d6	391.4/135	211	29	14	55	12.86
	7β-hydroxycholesterol-d7	392.3/159	81	33	14	55	13.75
	7-keto-cholesterol-d7	408.5/96	231	61	8	55	14.70

### 2.6 Liquid chromatography-tandem mass spectrometry (LC-MS/MS)

The analysis was done using liquid chromatography (LC, DIONEX UltiMate 3000, Thermo Scientific UK Ltd., Hemel Hempstead) on-line coupled to the ESI-QqLIT-MS/MS (QTRAP 5500, AB Sciex UK Ltd., Warrington). Samples (20 µl in 50% aqueous methanol with 1% formic acid) were separated on the reverse phase C18 column: NUCLEOSIL® C18, 100 mm, 5 µm pore size (Macherey-Nagel, Germany) using mobile phase (A) methanol: water:formic acid (70:30:0.1, v/v) and (B) isopropanol: methanol: formic acid (90:10:0.1, v/v) and a column temperature at 45 °C. Flow rate was maintained at 200 µl/min with the gradient as follows: 84% B from 0 to 7 min, 84–76% B from 7 to 11 min, 76–100% B from 11 to 25 min, 100% B 25–30 min, 100–84% B from 30 to 32 min, 84% B 32–48 min. Acquired data were processed using Analyst Software (version 1.7, AB Sciex).

## 3 Results

### 3.1 Development of the LC-MS/MS quantification method

**Fig. 1:**
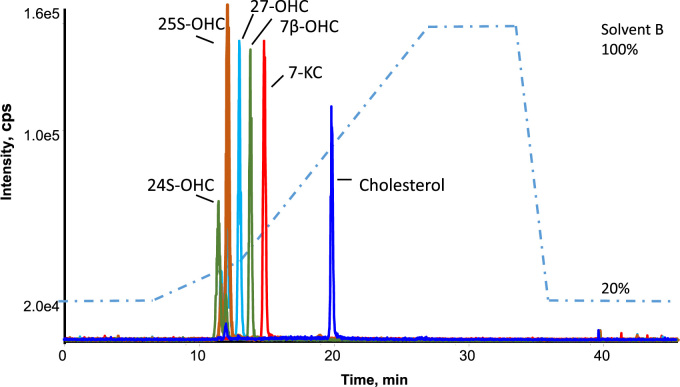
Chromatographic separation of oxysterols and cholesterol mixture (5 ng) in a 48 min run time. 24-hydroxycholesterol (385/161: RT = 11.43 min); 25-hydroxycholesterol (385/147: RT = 11.93 min); 27-hydroxycholesterol (385/161: RT = 12.88 min); 7β-hydroxycholesterol (385/81: RT = 13.80 min); 7keto-cholesterol (401/196: RT = 14.79 min); cholesterol (369/81: RT = 19.87 min).

A reproducible and sensitive LC-MS/MS method for the simultaneous quantitation of free 24S-OHC, 25-OHC, 27-OHC, 7β-OHC and 7-KC in plasma samples was developed in this study (Fig. 1). In contrast to previous methods [26], [27], a saponification step was not included. For the estimation of the process recovery the peak areas of five internal standards were compared before and after spiking to human plasma (Table S2). Results showed that IS enrichment with Oasis HLB prime cartridges gives the highest percentage recovery values for 24-OHCd7, 25-OHCd6, 27-OHCd6, 7β-OHCd7, and 7-KCd7 (77.60%, 81.80%, 80.65%, 72.04%, and 89.86% respectively) in plasma, compared to the Oasis HLB (72.11%, 74.11%, 76.70%, 41.58%, and 79.10%) and C18 cartridges (10.16%, 8.11%, 20.50%, 12.12%, and 15.66%). Polymeric chemistry with capabilities for both hydrophobic and polar retention are better than C18 reversed phase when it comes to enrichment.

The specificity and selectivity of the method was achieved by good chromatographic separation prior to sensitive mass spectrometry detection through careful design of Q1/Q3 *m*/*z* transition pairs (Table 2 and Table S3). Attention was given to chromatographic separation of oxysterol isomers; 24-OHC, 25-OHC and 27-OHC. Specifically, the identification of closely eluting 24S-OHC and 25-OHC have been achieved by narrow chromatographic peaks and analyte specific detection. An adequate separation was achieved using a multistep gradient, mobile phase composition (variation of isopropanol percentage in methanol) in combination with a careful selection of Q1/Q3 transition pairs.

### 3.2 Calibration curves, process recovery and reproducibility

Method validation was performed according to the “Center for Drug Evaluation and Research (CDER) Guidance for Industry: Bioanalytical Method Evaluation' (http://www.fda.gov/downloads/Drugs/Guidances/ucm070107.pdf) guidance. The linear dynamic range of the instrument was evaluated by measuring 17 different standard concentrations (0, 1, 5 fg; 1, 10, 100, 250, 500, 750 pg; 1, 2.5, 5, 10, 25, 50, 100, 250 and 500 ng per injection) for pooled authentic and deuterated standards (data not shown). Calibration curves used for quantification were designed for the each analyte, from the triplicate measurements of the control plasma and control plasma spiked with 10 different concentrations of standards (0.01, 0.1, 0.25, 0.5, 0.75, 1, 2.5, 5, 10 ng per injection). Linearity of the fit expressed by correlation coefficient R^2^ ≥ 0.99 indicated linearity (Fig. S1).

Limits of detection (LOD) and Limit of quantification (LOQ) are expressed as the analyte concentration corresponding to the sample blank value plus three and ten standard deviations respectively **(**Table 3**).** We determined intra- and inter- day precision and accuracy for the each analyte for the three different concentrations within linear dynamic range, namely for the concentrations corresponding to the lower (LLOQ), upper limits of quantification (ULOQ), and one concentration in between LLOQ and ULOQ (Table 4). Intraday precision was evaluated from the triplicate measurements of the mixture of authentic and deuterated standards, and was calculated for the three different standard concentrations within the linear dynamic range (1, 2 and 5 ng/ml). Calculated CVs for the each concentration point were within required range (< 15% in accordance with FDA guidelines), indicating proper precision for the intraday and interday reproducibility measurements. Process recovery (PR) was calculated for the three concentrations (from 0.5 to 5 ng/ml).

**Table 3:** **Precision data, calibration curves, linear dynamic range, detection limit and quantitation limit of the different sterols.** Limit of detection (LOD) is defined as the lowest detectable amount of analyte with a signal-to noise ratio (S/N) of 3:1, lower limit of quantification (LLOQ) is defined as the lowest quantifiable amount of analyte with S/N of 1:10 under experimental conditions, and both were determined for the each standard. The process recovery (the percentage of analyte change compared to the intensity measured for the pool of standards in methanol) was calculated for 1 ng of each authentic standard, and represented in percent with the relative standard deviation (SD).

Analyte	LLOQ, pg/ml	LOD, pg/ml	Process recovery ± SD, %
24 hydroxycholesterol	253	135	77.60 ± 10.5
25 hydroxycholesterol	122	24	81.80 ± 8.6
27 hydroxycholesterol	115	44	80.65 ± 10.4
7β-hydroxycholesterol	18	5	72.04 ± 11.6
7keto-cholesterol	39	12	79.86 ± 14.3

**Table 4:** Intraday, interday precision and CV of developed analytical procedure for quantification of 5 authentic standards (n = 3).

Analyte	Concentration (ng/ml)	Inter day			Intraday		
		Measured (ng/ml)	CV %	% error	Measured (ng/ml)	CV %	% error
24S hydroxycholesterol	0.1	0.092	12.8	8.70	0.11	2.7	9.09
	1	0.93	4	7.53	0.98	8.1	2.04
	50	45	6.4	11.11	51	3.8	1.96
25 hydroxycholesterol	0.1	0.11	12	9.09	0.11	15	9.09
	1	1.08	10	7.41	1.01	3.4	0.99
	50	53	4.5	5.66	49	8.6	2.04
27 hydroxycholesterol	0.1	0.096	7.0	4.17	0.1	5.3	0.00
	1	1.05	5.2	4.76	0.99	6.1	1.01
	50	48	3.7	4.17	51	0.7	1.96
7β-hydroxycholesterol	0.1	0.11	8.1	9.09	0.98	10.3	2.04
	1	1.1	5.7	9.09	1.2	2.7	16.67
	50	52	1.2	3.85	48	0.8	4.17
7keto-cholesterol	0.1	0.097	10.4	3.09	0.099	6.3	1.01
	1	1.03	7.1	2.91	1.01	4.1	0.99
	50	51	6.4	1.96	51	1.2	1.96

### 3.3 Quantification of oxysterols in hypercholesterolaemic patients

Plasma cholesterol levels were significantly different between the two groups at baseline (control group = 4.08 ± 0.18 mM; hypercholesterolaemic group = 6.72 ± 0.78 mM; p < 0.001, Table 1). After 40 mg simvastatin treatment per day for 3 months, the subjects with hypercholesterolaemia showed a 35% reduction in cholesterol levels and there was no significant difference observed in other measurements between the two groups.

All five oxysterols measured in this study were higher in statin-naive hypercholesterolaemic men compared to age-matched control subjects at baseline; 24S-OHC, 25-OHC, 7β-OHC and 7-KC were 2, 8, 1.5, 45 and 33 fold higher (Table 1). Absolute concentrations of free radical dependent oxysterols, 7β-OHC and 7-KC were significantly increased (P < 0.0001) in hypercholesterolaemic men at baseline and after 3 months intervention with simvastatin, oxysterol concentrations in plasma were similar to those of healthy subjects.

**Fig. 2:**
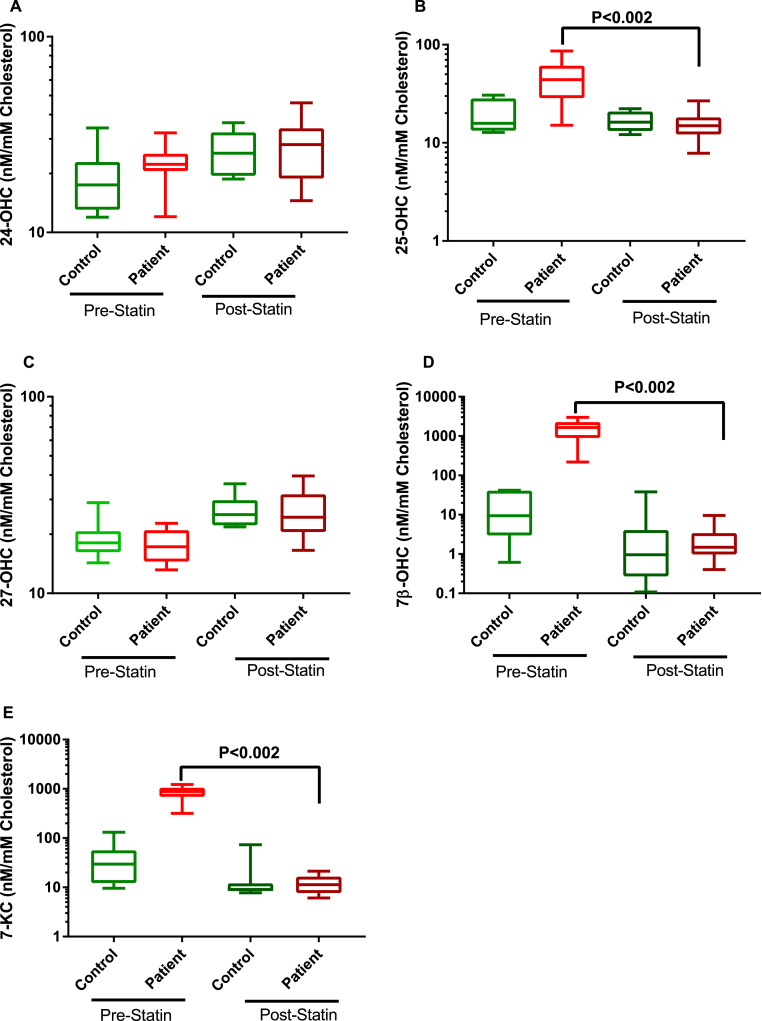
Free oxysterol concentrations adjusted to free cholesterol in plasma from hypercholesterolaemic patients before and after Simvastatin intervention (n = 10 in each group). Box plots show the plasma oxysterols; (A) 24S-OHC (B) 25-OHC (C) 27-OHC (D) 7β-OHC (E) 7-KC. Data was analysed by Wilcoxon matched pair *t-*test.

Owing to the reduction in total cholesterol with intervention we normalised the calculated oxysterol measurements (nM) to total plasma cholesterol concentration (mM) and the data has been expressed as a ratio, nM of oxysterols: mM of total cholesterol. Levels of enzymatically produced 24S-OHC and 27-OHC were not significantly different between any groups irrespective of statin intervention (Fig. 2). 25-OHC, 7β-OHC and 7-KC levels are significantly higher in men with hypercholesterolaemia even after correcting for cholesterol. Intake of 40 mg simvastatin for 3 months reduced the level of these oxysterols to the levels recorded in healthy control subjects.

## 4 Discussion

The method described here for the simultaneous detection of five oxysterols has been optimised for analysis of non-esterified plasma oxysterols through a simple solid phase preparative procedure. The current method avoids derivatisation steps and uses Oasis HLB prime cartridges for fast sample preparation. With excellent recovery, we have been able to analyse oxysterols that normally circulate at concentrations which are six orders of magnitude lower than the parent cholesterol. For the first time, this method has been applied to study the effects of three months of statin intervention on plasma oxysterol concentrations in midlife men with high plasma cholesterol. We showed that the free radical-dependent oxysterols (7-KC, 7β-OHC and 25-OHC) were circulating in people with asymptomatic hypercholesterolaemia at up to 45 times the concentrations that could be observed in men with normal plasma cholesterol and that the significance of the effect was maintained either before or after correction for differences in plasma cholesterol concentration. Simvastatin treatment for three months reduced oxysterol concentrations to those seen in healthy men with normal lipid profiles.

The enzymatically produced oxysterols, 24S- and 27-OHC were not different between the population who presented with statin-naïve hypercholesterolaemia and the age-matched control subjects. Furthermore, there was no effect of statins on 24S- and 27-OHC in hypercholesterolaemic subjects over three months either before or after correction for a reduction in plasma cholesterol concentration. In contrast, a previous study in patients with coronary artery stenosis reported that statins reduced total oxysterol concentrations from 24 nM to 15 nM; these authors used an ELISA method that did not specify which of the oxysterols was being analysed [25].

Also, Vega et al., showed that plasma concentrations of 24-OHC and 7-OHC but not 27-OHC were higher in dementia than in healthy controls and may be reduced by simvastatin [28], [29], [30], [31]. They reported levels of 60 ng/ml of 24-OHC in dementia patients whereas we detected levels that were 50% lower in midlife men without overt disease. The previous studies used a method that measured both free and esterified oxysterols, whereas our method focussed on free oxysterols. In the present study, we wished to focus on free oxysterols that are exposed in plasma rather than being held in the core of lipoproteins. Free oxysterols are biologically active and have been shown to bind to cysteine rich domains of Smoothened and enable maximal activation of the hedgehog signalling pathway by specific receptor ligands [32]. This pathway is emerging as an important target for cardiovascular disease and cancer [33].

Autoxidised cholesterols, including 7-KC and 7β-OHC have been shown to affect the bioavailability of other reactive oxygen and nitrogen species, by chemical interaction with nitric oxide. In addition, oxysterols easily diffuse into membranes where they affect receptor and enzyme function [34]; furthermore, 7-KC promotes translocation of cytosolic NADPH oxidase components to the membrane in neutrophils and enhances rapid reactive oxygen species production [35] are well-known activators of NADPH oxidase.

To maintain cell homeostasis, cholesterol concentrations are tightly regulated. Oxysterols play a role in maintaining cholesterol homeostasis acting as ligands for SREBP2 and downregulating endogenous cholesterol synthesis via decreased expression of HMG CoA reductase in a similar way to the mechanism of statin actions. The fact that both cholesterol and autoxidised oxysterols were reduced by statins in people with hypercholesterolaemia but not oxysterols themselves, suggests an inability of the homeostatic mechanism to manage excessive cholesterol arising from dietary intake although the pharmacological action of statins on HMG CoA reductase was effective.

Vascular risk factors and comorbidities are extremely frequent in cognitive impairment with and without dementia, but it is difficult to assess the causal role of cardiometabolic factors in dementia onset. This is due to the fact that the pathophysiological mechanisms that are likely to confer chronological primacy in neurodegeneration occur decades prior to the clinical onset of overt symptoms of cognitive decline. Therefore, the main focus of the present study was evaluating men with high cholesterol in midlife but without any overt signs of disease, in order to understand whether any of the differences in plasma oxysterols observed in AD patients are already present in hypercholesterolaemic men in midlife and constitute therefore a risk for dementia development. The link between high cholesterol and later development of dementia is less strong in women [36]. It is known that oestrogenic hormones can themselves affect sterol metabolism and this may in part explain the protection that women experience from vascular disease.

To further understand any mechanistic relationship between plasma cholesterol and later development of dementia, others have investigated the effect of simvastatin on levels of the toxic protein phospho-tau in the cerebrospinal fluid of cognitively normal adults aged 45–60 years and a positive relationship between tau and plasma LDL was observed [36]. This suggests that there is a relationship between neurotoxic peptides and cholesterol in midlife. However, when statins were prescribed to patients with pre-existing dementia, there was no benefit for cognition [37]. Taken together, this evidence suggests that modification of cholesterol metabolism in mid- rather than later life may reduce risk for dementia.

In conclusion, our simple method for determining five oxysterols in plasma has shown that autoxidation products of cholesterol are up to 45 times greater in the plasma of asymptomatic, hypercholesterolaemic men and that within 3 months, autoxidised oxysterol concentration was normalised by simvastatin treatment.

## References

[1] Gottesman R.F., Albert M.S., Alonso A., Coker L.H., Coresh J., Davis S.M. (2017). Associations between midlife vascular risk factors and 25-year incident dementia in the atherosclerosis risk in communities (ARIC) cohort. JAMA Neurol..

[2] Perna L., Mons U., Rujescu D., Kliegel M., Brenner H. (2016). Apolipoprotein E e4 and cognitive function: a modifiable association results from two independent cohort studies. Dement. Geriatr. Cogn. Disord..

[3] Alonso A., Jacobs D.R., Menotti A., Nissinen A., Dontas A., Kafatos A. (2009). Cardiovascular risk factors and dementia mortality: 40 years of follow-up in the Seven Countries Study. J. Neurol. Sci..

[4] Kivipelto M., Ngandu T., Laatikainen T., Winblad B., Soininen H., Tuomilehto J. (2006). Risk score for the prediction of dementia risk in 20 years among middle aged people: a longitudinal, population-based study. Lancet Neurol..

[5] Polidori M.C., Pientka L., Mecocci P. (2012). A review of the major vascular risk factors related to Alzheimer's disease. J. Alzheimer's. Dis.: JAD.

[6] Corrao G., Ibrahim B., Nicotra F., Zambon A., Merlino L., Pasini T.S. (2013). Long-term use of statins reduces the risk of hospitalization for dementia. Atherosclerosis.

[7] Cramer C., Haan M.N., Galea S., Langa K.M., Kalbfleisch J.D. (2008). Use of statins and incidence of dementia and cognitive impairment without dementia in a cohort study. Neurology.

[8] Dias I.H., Polidori M.C., Li L., Weber D., Stahl W., Nelles G. (2014). Plasma levels of HDL and carotenoids are lower in dementia patients with vascular comorbidities. J. Alzheimer's. Dis.: JAD.

[9] Polidori M.C., Mattioli P., Aldred S., Cecchetti R., Stahl W., Griffiths H. (2004). Plasma antioxidant status, immunoglobulin g oxidation and lipid peroxidation in demented patients: relevance to Alzheimer disease and vascular dementia. Dement. Geriatr. Cogn. Disord..

[10] Dias H.K.I., Brown C.L.R., Polidori M.C., Lip G.Y.H., Griffiths H.R. (2015). LDL-lipids from patients with hypercholesterolaemia and Alzheimer's disease are inflammatory to microvascular endothelial cells: mitigation by statin intervention. Clin. Sci..

[11] Dias I.H., Mistry J., Fell S., Reis A., Spickett C.M., Polidori M.C. (2014). Oxidized LDL lipids increase beta-amyloid production by SH-SY5Y cells through glutathione depletion and lipid raft formation. Free Radic. Biol. Med..

[12] Olkkonen V.M., Béaslas O., Nissilä E. (2012). Oxysterols and their cellular effectors. Biomolecules.

[13] Björkhem I., Lütjohann D., Breuer O., Sakinis A., Wennmalm Å. (1997). Importance of a novel oxidative mechanism for elimination of brain cholesterol: turnover of cholesterol and 24(S)-hydroxycholesterol in rat brain as measured with 18o2 techniques in vivo and in vitro. J. Biol. Chem..

[14] Okabe A., Urano Y., Itoh S., Suda N., Kotani R., Nishimura Y. (2013). Adaptive responses induced by 24S-hydroxycholesterol through liver X receptor pathway reduce 7-ketocholesterol-caused neuronal cell death. Redox Biol..

[15] Mateos L., Ismail M.A., Gil-Bea F.J., Leoni V., Winblad B., Bjorkhem I. (2011). Upregulation of brain renin angiotensin system by 27-hydroxycholesterol in Alzheimer's disease. J. Alzheimer's. Dis..

[16] Liu Q., An Y., Yu H., Lu Y., Feng L., Wang C. (2016). Relationship between oxysterols and mild cognitive impairment in the elderly: a case-control study. Lipids Health Dis..

[17] Virginio V.W., Nunes V.S., Moura F.A., Menezes F.H., Andreollo N.A., Rogerio F. (2015). Arterial tissue and plasma concentration of enzymatic-driven oxysterols are associated with severe peripheral atherosclerotic disease and systemic inflammatory activity. Free Radic. Res..

[18] Rosa-Fernandes L., Maselli L.M.F., Maeda N.Y., Palmisano G., Bydlowski S.P. (2017). Outside-in, inside-out: proteomic analysis of endothelial stress mediated by 7-ketocholesterol. Chem. Phys. Lipids.

[19] Prunet C., Petit J.M., Ecarnot-Laubriet A., Athias A., Miguet-Alfonsi C., Rohmer J.F. (2006). High circulating levels of 7beta- and 7alpha-hydroxycholesterol and presence of apoptotic and oxidative markers in arterial lesions of normocholesterolemic atherosclerotic patients undergoing endarterectomy. Pathol.-Biol..

[20] Diczfalusy U. (2013). On the formation and possible biological role of 25-hydroxycholesterol. Biochimie.

[21] Choi S.H., Sviridov D., Miller Y.I. (2017). Oxidized cholesteryl esters and inflammation. Biochim. Biophys. Acta.

[22] Chen J.H., Peng D.Q. (2015). Is 25-hydroxycholesterol the interplay of statins and inflammation?. Int. J. Cardiol..

[23] van Poppel G., van de Vijver L.P., Kosmeyer-Schuil T., Johanns E.S., Kardinaal A.F., van de Bovenkamp P. (1997). Plasma oxysterols and angiographically determined coronary atherosclerosis: a case control study. Biomark.: Biochem. Indic. Expo. Response Susceptibility Chem..

[24] Thelen K.M., Lütjohann D., Vesalainen R., Janatuinen T., Knuuti J., von Bergmann K. (2006). Effect of pravastatin on plasma sterols and oxysterols in men. Eur. J. Clin. Pharmacol..

[25] Pordal A.H., Hajmiresmail S.J., Assadpoor-Piranfar M., Hedayati M., Ajami M. (2015). Plasma oxysterol level in patients with coronary artery stenosis and its changes in response to the treatment with atorvastatin. Med. J. Islam. Repub. Iran..

[26] Karuna R., von Eckardstein A., Rentsch K.M. (2009). Dopant assisted-atmospheric pressure photoionization (DA-APPI) liquid chromatography–mass spectrometry for the quantification of 27-hydroxycholesterol in plasma. J. Chromatogr. B..

[27] Baila-Rueda L., Cenarro A., Cofan M., Orera I., Barcelo-Batllori S., Pocovi M. (2013). Simultaneous determination of oxysterols, phytosterols and cholesterol precursors by high performance liquid chromatography tandem mass spectrometry in human serum. Anal. Methods.

[28] van de Kraats C., Killestein J., Popescu V., Rijkers E., Vrenken H., Lutjohann D. (2014). Oxysterols and cholesterol precursors correlate to magnetic resonance imaging measures of neurodegeneration in multiple sclerosis. Mult. Scler..

[29] Vega G.L., Weiner M., Kolsch H., von Bergmann K., Heun R., Lutjohan D. (2004). The effects of gender and CYP46 and apo E polymorphism on 24S-hydroxycholesterol levels in Alzheimer's patients treated with statins. Curr. Alzheimer Res..

[30] Vega G.L., Weiner M.F. (2007). Plasma 24S hydroxycholesterol response to statins in Alzheimer's disease patients: effects of gender, CYP46, and ApoE polymorphisms. J. Mol. Neurosci..

[31] Vega G.L., Weiner M.F., Lipton A.M., Von Bergmann K., Lutjohann D., Moore C. (2003). Reduction in levels of 24S-hydroxycholesterol by statin treatment in patients with Alzheimer disease. Arch. Neurol..

[32] Nedelcu D., Liu J., Xu Y., Jao C., Salic A. (2013). Oxysterol binding to the extracellular domain of smoothened in Hedgehog signaling. Nat. Chem. Biol..

[33] Redmond E.M., Guha S., Walls D., Cahill P.A. (2011). Investigational Notch and Hedgehog inhibitors – therapies for cardiovascular disease. Expert Opin. Investig. Drugs.

[34] Gargiulo S., Testa G., Gamba P., Staurenghi E., Poli G., Leonarduzzi G. (2017). Oxysterols and 4-hydroxy-2-nonenal contribute to atherosclerotic plaque destabilization. Free Radic. Biol. Med..

[35] Alba G., Reyes-Quiróz M.E., Sáenz J., Geniz I., Jiménez J., Martín-Nieto J. (2016). 7-Keto-cholesterol and 25-hydroxy-1 cholesterol rapidly enhance ROS production in human neutrophils. Eur. J. Nutr..

[36] Li G., Mayer C.L., Morelli D., Millard S.P., Raskind W.H., Petrie E.C. (2017). Effect of simvastatin on CSF Alzheimer disease biomarkers in cognitively normal adults. Neurology.

[37] McGuinness B., Craig D., Bullock R., Malouf R., Passmore P. (2014). Statins for the treatment of dementia. Cochrane Database Syst. Rev..

