# Supplementary material for: Simvastatin reduces circulating oxysterol levels in men with hypercholesterolaemia
Source: Redox Biol. 2018 Feb 17;16:139–45. doi: 10.1016/j.redox.2018.02.014 (PMC5952874; doi:10.1016/j.redox.2018.02.014)
Supplement: Supplementary file 1 — Supplementary material [file mmc1.docx]

**Supplementary figures and tables**

**Figure S1:** Linear dynamic range of deuterated oxysterol standards; (A) 24S-OHC-d7 (B) 25-OHC-d6 (C) 27-OHC-d6 (D) 7β-OHC-d7 (E) 7-KCd7. Plasma (70 µl) was used with and without spiking with methanolic solution of all authentic and internal standards (0, 0.1, 0.25, 0.5, 0.75, 1, 2.5, 5, 10 ng) in triplicate to generate standard curves.

| **Analyte** | **RT, min** | **Oasis HLB Prime (%)** | **Oasis HLB (%)** | **Discovery DSC-18 (%)** |
| --- | --- | --- | --- | --- |
| 24S-OHC-d7 | 15.51 | 77.60 | 72.11 | 10.16 |
| 25-OHC-d6 | 15.79 | 81.80 | 74.11 | 8.11 |
| 27-OHC-d6 | 16.80 | 80.65 | 76.70 | 20.50 |
| 7β-OHC-d7 | 17.36 | 72.04 | 41.58 | 12.12 |
| 7-KCd7 | 18.20 | 79.86 | 79.10 | 15.66 |

**Table S1**: Comparison of percentage process recoveries for 24-OHCd7, 25-OHCd6, 27-OHCd6, 7β-OHCd7, 7-KCd7 in plasma after enrichment with Oasis HLB, Oasis HLB Prime and Discovery DSC-18 columns.

| **Analyte** | Number of freeze-thaw cycles, percentage recovery (%) | | | |
| --- | --- | --- | --- | --- |
|  | 1 | 2 | 3 | 4 |
| 24SOHC | 75.3 | 65.3 | 41.3 | 21.4 |
| 25OHC | 72.6 | 68.1 | 52.6 | 12.3 |
| 27OHC | 71.8 | 59.3 | 48.2 | 23.6 |
| 7βOHC | 68.2 | 61.3 | 52.3 | 31.3 |
| 7-KC | 72.7 | 52.1 | 52.6 | 43.1 |

**Table S2**: The effect of the freeze-thaw cycles on the stability of oxysterols. One plasma sample was spiked with a mix of 1ng authentic standards was frozen at -80^0^C. Aliquots of 70 µl were taken through 1-4 freeze-thaw cycles. Lipids were extracted and analysed for levels of free oxysterols. Percentage recovery was calculated against a plasma sample spiked with mix of authentic standards spiked at 1ng on the day.

| ID | Q1 mass (Da) | Q3 mass (Da) | Dwell Time (msec) | DP (V) | CE (V) | CXP (V) |
| --- | --- | --- | --- | --- | --- | --- |
| Chl_1 | 369.2 | 81 | 100 | 196 | 45 | 14 |
| Chl_3 | 369.2 | 109 | 100 | 211 | 37 | 14 |
| Chl_4 | 369.2 | 135 | 100 | 131 | 25 | 12 |
| 24-OHC_1 | 385.4 | 147 | 100 | 191 | 33 | 12 |
| 24-OHC_2 | 385.3 | 161 | 100 | 166 | 29 | 24 |
| 24-OHC_3 | 385.3 | 81 | 100 | 181 | 65 | 14 |
| 24-OHC_4 | 385.3 | 95 | 100 | 161 | 41 | 14 |
| 25-OHC_1 | 367.3 | 81 | 100 | 221 | 53 | 12 |
| 25-OHC_2 | 367.3 | 95 | 100 | 221 | 41 | 14 |
| 25-OHC_3 | 367.3 | 133 | 100 | 216 | 43 | 16 |
| 25-OHC_4 | 367.3 | 159 | 100 | 211 | 39 | 22 |
| 25-OHC_5 | 367.3 | 161 | 100 | 211 | 29 | 12 |
| 27-OHC_1 | 385.4 | 161 | 100 | 181 | 33 | 14 |
| 27-OHC_2 | 385.3 | 147 | 100 | 161 | 33 | 20 |
| 7β-OHC_1 | 385.4 | 81 | 100 | 216 | 53 | 8 |
| 7β-OHC_2 | 385.4 | 159 | 100 | 176 | 37 | 10 |
| 7-KC_1 | 401.4 | 69 | 100 | 136 | 51 | 8 |
| 7-KC_2 | 401.4 | 95 | 100 | 196 | 41 | 16 |
| 7-KC_3 | 401.4 | 121 | 100 | 120 | 45 | 36 |
| 7-KC_4 | 401.4 | 175 | 100 | 131 | 39 | 6 |
| 24S-OHC-d7_1 | 392.4 | 135 | 100 | 196 | 35 | 6 |
| 24S-OHC-d7_2 | 392.4 | 161 | 100 | 191 | 37 | 16 |
| 24S-OHC-d7_3 | 392.4 | 283 | 100 | 206 | 17 | 14 |
| 25-OHC-d6_1 | 391.6 | 133 | 100 | 41 | 29 | 18 |
| 25-OHC-d6_2 | 391.6 | 161 | 100 | 121 | 33 | 18 |
| 27-OHC-d6_1 | 391.4 | 135 | 100 | 211 | 29 | 14 |
| 27-OHC-d6_2 | 391.4 | 161 | 100 | 201 | 27 | 12 |
| 27-OHC-d6_3 | 391.4 | 175 | 100 | 196 | 31 | 22 |
| 27-OHC-d6_4 | 391.4 | 257 | 100 | 181 | 23 | 55 |
| 7-KCd7_1 | 408.5 | 96 | 100 | 231 | 61 | 8 |
| 7-KCd7_2 | 408.5 | 107 | 100 | 256 | 55 | 28 |
| 7-KCd7_3 | 408.5 | 157 | 100 | 221 | 43 | 22 |
| 7β-OHC-d7_1 | 392.3 | 105 | 100 | 101 | 71 | 10 |
| 7β-OHC-d7_2 | 392.3 | 159 | 100 | 81 | 33 | 14 |
| 7β-OHC-d7_3 | 392.3 | 374 | 100 | 161 | 19 | 28 |

**Table S3:** Total Multiple reaction monitoring (MRM) parameters (Q1/Q3 transition pair; declustering potential (DP); collision energy (CE); exit quadrupole potential, (CXP), retention times) used in the method.
